# Sorafenib-related generalized eruptive keratoacanthomas (Grzybowski syndrome): a case report

**DOI:** 10.1186/s13256-021-03037-4

**Published:** 2021-09-21

**Authors:** M. Nazim Abbas, Wei Son Tan, Ganessan Kichenadasse

**Affiliations:** 1 Flinders Centre for Innovation in Cancer, Flinders Medical Centre, Adelaide, South Australia Australia; 2 Flinders University, Adelaide, South Australia Australia

**Keywords:** Sorafenib, Grzybowski syndrome, Hepatocellular carcinoma

## Abstract

**Background:**

Sorafenib is an oral multikinase inhibitor that targets Raf serine/threonine receptor tyrosine kinases and inhibits tumor cell growth and angiogenesis. Cutaneous toxicities of sorafenib are common, including cutaneous eruptions (such as truncal erythema and seborrheic-dermatitis-like changes) and hand–foot syndrome. Keratoacanthomas and squamous cell carcinomas have been reported previously; however, we report a case of multiple eruptive keratoacanthomas in the form of Grzybowski syndrome after initiation of sorafenib.

**Case presentation:**

We report a 63-year-old Caucasian male who developed multiple cutaneous eruptive keratoacanthomas after starting sorafenib 400 mg twice daily. He had a known history of hepatitis-C-related cirrhosis and hepatocellular carcinoma, and previously had actinic keratosis and skin squamous cell carcinoma excision. Approximately two and a half months after starting sorafenib, the patient initially developed two lesions, one on each forearm, and after excision, these lesions demonstrated histological features of squamous cell carcinoma. One month later, the patient presented with approximately 48 new skin lesions of varying size on the back, bilateral upper limbs, and face requiring excisional biopsy of a large number of these lesions. Histopathology showed eruptive invasive keratoacanthomas (Grzybowski syndrome). Sorafenib was temporarily stopped and subsequently restarted at a lower dose. Acitretin 25 mg daily was commenced after few weeks, and no further keratoacanthomas developed during his treatment.

**Conclusions:**

We report a unique case of sorafenib-associated Grzybowski syndrome. Temporary interruption and dose reduction of sorafenib and use of acitretin appeared to prevent further development of keratoacanthomas.

## Background

Sorafenib is an oral small-molecule antineoplastic agent targeting multiple protein kinases and has been approved for the treatment of metastatic renal cell carcinoma and hepatocellular carcinoma. Sorafenib is an inhibitor of the serine/threonine kinase RAF pathway (inhibiting BRAF and RAF-1). Sorafenib inhibits proangiogenic and proliferative tyrosine kinases, including vascular endothelial growth factor (VEGF) 1/2/3, platelet-derived growth factor (PDGFR), c-kit, Flt-3, and RET [1]. Common cutaneous toxicities of sorafenib include rash, hand–foot skin reactions, subungual splinter hemorrhages, scalp dysesthesia, erythema of the face/scalp, stomatitis, and alopecia. Increasing observations of cutaneous squamous cell carcinoma and keratoacanthoma (KA) in association with sorafenib have been reported [2–4]; however, generalized eruptive keratoacanthomas (Grzybowski syndrome) related to sorafenib has not been previously identified. We report a patient who developed multiple keratoacanthomas consistent with Grzybowski syndrome after initiation of sorafenib therapy.

## Case presentation

A 63-year-old Caucasian male with known hepatocellular carcinoma presented with multiple cutaneous eruptive keratoacanthomas approximately three and half months after starting sorafenib 400 mg twice daily.

Past medical history included hepatitis C genotype 3A related hepatic cirrhosis (Child–Pugh class A), chronic obstructive pulmonary disease, portal and hepatic vein thrombosis, hypertension, and motorbike accident leading to fourth cervical vertebral fracture. Regular medications included perindopril, amlodipine, docusate with senna, and cholecalciferol.

The patient had a known history of actinic keratosis and cutaneous squamous cell carcinomas (first diagnosed more than 4 years before starting sorafenib) and had two well-differentiated squamous cell carcinomas completely excised on his right cheek about 2 months before the diagnosis of hepatocellular carcinoma (HCC). The patient had Fitzpatrick type II skin, and he had no peripheral stigmata of chronic liver disease particularly skin-related manifestations, including spider naevi, palmar erythema, or jaundice before starting sorafenib, although he had several skin tattoos.

The patient initially developed two hyperkeratotic erythematous nodules on the right and left forearm (approximately two and half months after starting sorafenib) with rapid growth over 1–2 months. Clinically, these lesions were assessed as keratoacanthomas; however, both lesions were excised with clear margins, and histology demonstrated features of squamous cell carcinoma with invasion into the deep reticular dermis.

**Fig. 1:**
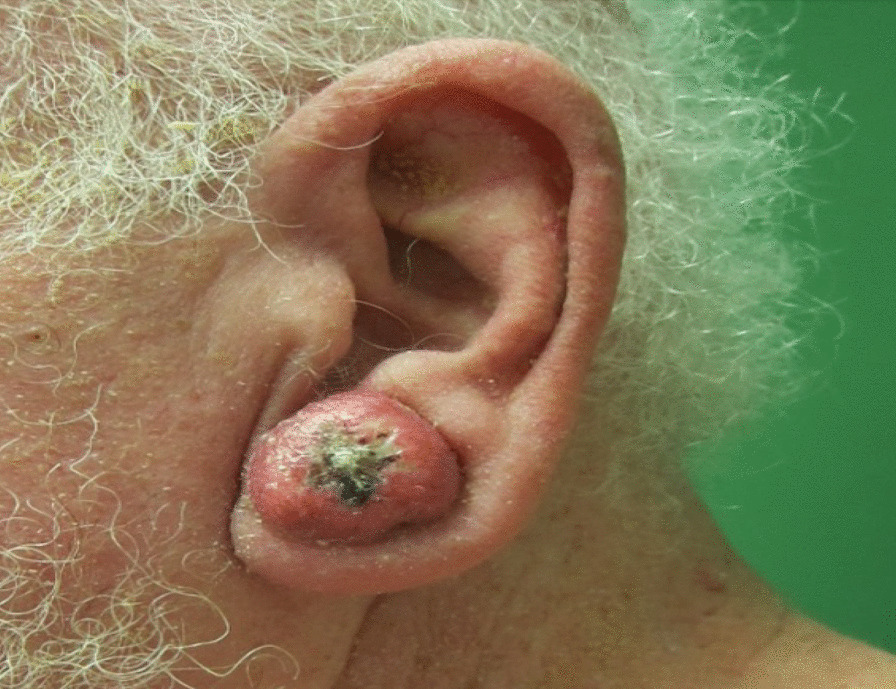
Left ear lobe keratoacanthoma—round, flesh-colored nodules with sharply sloping borders and a characteristic central crater containing keratinous material

**Fig. 2:**
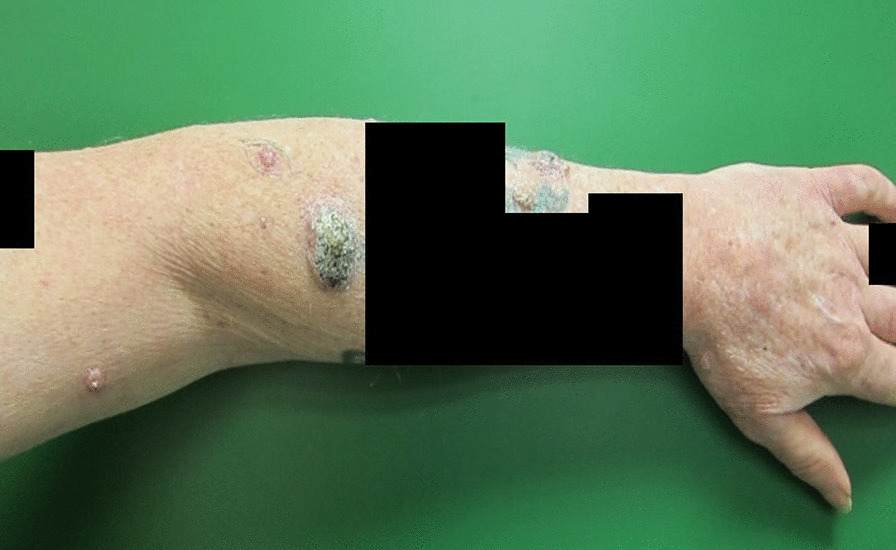
Multiple keratoacanthomas on left forearm

One month later (three and half months after starting sorafenib), the patient presented with approximately 48 new skin lesions of varying size on the left ear lobe (Figure 1), left lateral cheek, right side of the nose, central back, left shoulder, left arm and forearm (Figure 2), right wrist, right arm, and forearm. These lesions appeared on sun-damaged skin and clinically correlated with multiple keratoacanthomas, appearing as multiple, firm, rounded nodules with central crateriform architecture and rapid growth over a month.

**Fig. 3:**
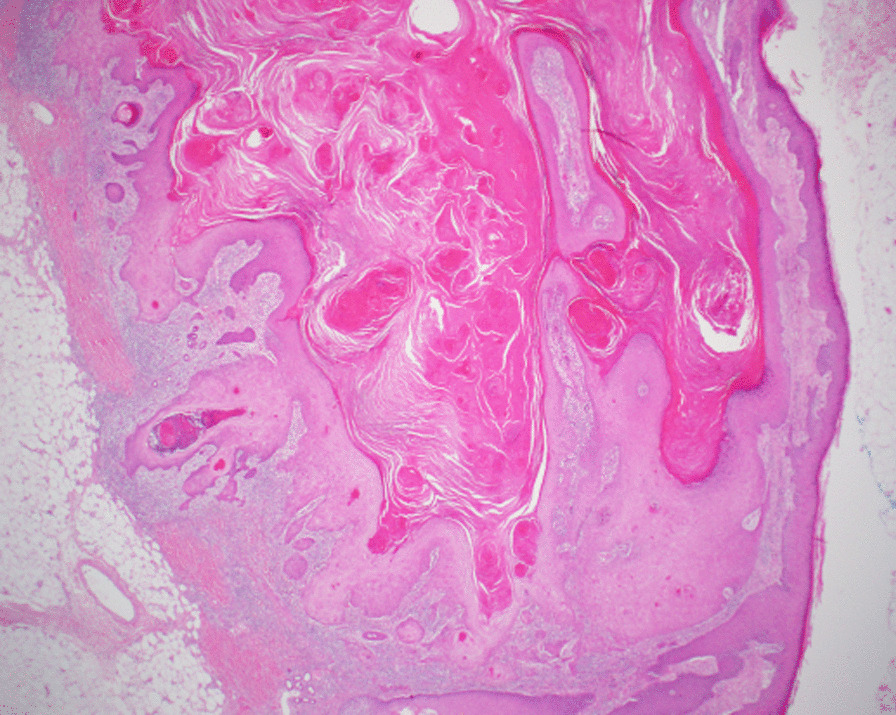
Low-power view (×2 magnification) of the left distal forearm skin excision, showing the edge of a crateriform squamous proliferation with histological features of keratoacanthoma

**Fig. 4:**
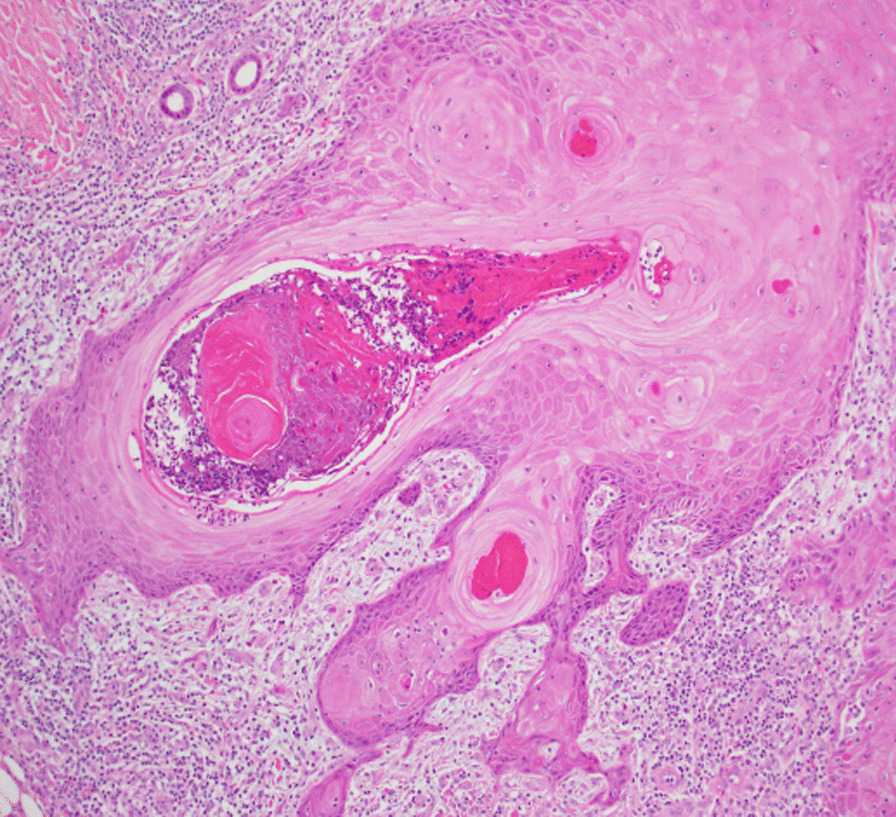
Higher-power objective view (×10) showing proliferating squamous epithelium. The lesion cells show glassy cytoplasm and lack cytological atypia or mitotic activity. The proliferating cells lie within a loose fibroinflammatory stroma

**Fig. 5:**
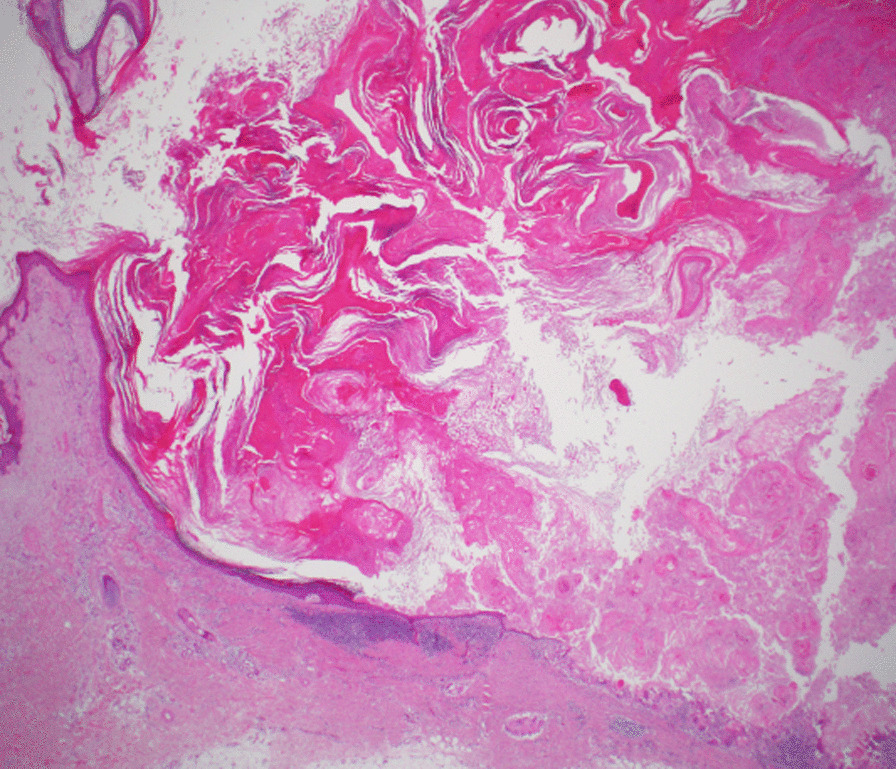
Images from the left proximal forearm lesion, which is in a later stage of evolution and is partly regressed. There is a residual attenuated layer of squamous epithelium that rests upon a fibrous scar. A foreign body giant cell reaction to keratin is also noted. Image is taken using the ×4 objective lens

The patient subsequently underwent complete excision of 15 lesions as surgical resection of all the lesions was not possible due to a significant number of lesions. Histopathology confirmed that the lesions were eruptive invasive keratoacanthomas (Grzybowski syndrome) (Figs. 3, 4, 5). Sorafenib was temporarily stopped for 3 weeks. No new lesions developed during this time; however, there was no spontaneous resolution of the existing lesions. After having a discussion with the patient and given the biochemical (reduction in alpha-fetoprotein levels) and the radiological response, it was decided to restart sorafenib at a lower dose (200 mg twice daily). Acitretin 25 mg daily was commenced for the remaining lesions at the same time when sorafenib was restarted, and no further keratoacanthomas developed during his treatment. The size of the remaining lesions was subsequently reduced with ongoing acitretin treatment. The patient developed pulmonary embolism and multiple cerebral embolic infarcts approximately 6 months later and died. No further lesions appeared or required surgical management, and the residual lesions reduced in size during this period. Sorafenib and acitretin were discontinued 2 weeks before his death.

## Discussion

Keratoacanthomas are cutaneous 1–2 cm squamoproliferative nodules commonly characterized by periods of rapid growth, lesion stability, and spontaneous resolution. Although they are usually considered benign, it is difficult to clinically and histologically differentiate keratoacanthoma from squamous cell carcinoma, and the current treatment recommendation is an excisional biopsy of the lesion [5].

Keratoacanthomas can appear as solitary lesions or in rare cases manifest as multiple eruptive lesions. Different patterns of multiple eruptive keratoacanthomas include familial keratoacanthomas (Ferguson–Smith type), which are characterized by the sudden appearance and then spontaneous regression of squamous cell carcinomas or keratoacanthomas. In Muir–Torre syndrome, multiple keratoacanthomas appear on the skin, and it is also associated with sebaceous skin tumors and gastrointestinal and genitourinary carcinomas. Acute-onset generalized eruptive keratoacanthomas (Grzybowski syndrome) is an extremely rare condition with approximately 30 cases reported worldwide [6].

Pathophysiology of generalized eruptive keratoacanthomas (Grzybowski syndrome) is poorly understood. There is no diagnostic criterion for Grzybowski syndrome; however, the condition manifests as a sudden eruption, and subsequent progressive onset of hundreds to thousands of keratoacanthomas, with oral retinoids being the preferred treatment option [7]. Keratoacanthomas have been previously reported in association with sorafenib treatment with possible involvement of RET proto-oncogene, PDGFR, and RAS pathway inhibition leading to epithelial proliferation with reduced antitumor immunity [8].

Multiple keratoacanthomas associated with sorafenib have been reported previously in the case reports. Jantzem *et al*. [9] reported multiple keratoacanthomas in a patient with renal cell carcinoma, and the keratoacanthomas responded to 5% fluorouracil cream and dose reduction of sorafenib. The lesions eventually resolved after stopping sorafenib. Kong *et al*. [2] reported three cases with the number of lesions ranging between 1 and 9 and consistent with keratoacanthomas. These lesions responded well to temporary sorafenib dose interruption or dose reduction. Marquez *et al*. [10] reported a patient with ovarian cancer who developed two keratoacanthomas requiring surgical excision nearly 3 months after starting sorafenib in combination with carboplatin and tamoxifen. The patient developed two more keratoacanthomas after a 2-month interval and underwent further surgical resection. Six keratoacanthomas appeared a few months later while sorafenib was continued and bexarotene was used to treat the lesions. After 3 months of treatment with bexarotene, five lesions disappeared and one lesion significantly reduced in size. Mesarch *et al*. [11] reported a patient with renal cell carcinoma who developed multiple squamous cell carcinomas with keratoacanthomatous features about 4 weeks after starting sorafenib. These lesions were treated with excision, electrodesiccation, curettage, cryotherapy, and sorafenib dose reduction, which resulted in decrease in the number of lesions. Sorafenib was subsequently stopped, and significant improvement was observed within a few weeks.

We reported a unique case of sorafenib-associated multiple eruptive keratoacanthomas in the form of Grzybowski syndrome. We also explored other possible causes for the development of skin lesions and considered the possibility of concomitant medications and their potential interactions. We found no previously reported significant interaction between these drugs that could potentially increase the risk of skin lesions particularly in the setting of chronic liver disease (Child–Pugh A cirrhosis). There was a temporal correlation between the commencement of sorafenib and the development of keratoacanthomas, and we, therefore, considered sorafenib as the likely cause for keratoacanthomas. Temporary interruption and dose reduction of sorafenib and use of acitretin appeared to prevent further development of keratoacanthomas.

The occurrence of such eruptive keratoacanthomas in the form of Grzybowski syndrome associated with sorafenib has not previously been reported. This case highlights the need for regular monitoring of the skin of patients commenced on sorafenib therapy.

## Conclusion

We reported a unique case of sorafenib-associated Grzybowski syndrome, and we observed that temporary interruption and dose reduction of sorafenib and use of acitretin could prevent further development of keratoacanthomas.

## Data Availability

The datasets during and/or analyzed during the current study available from the corresponding author on reasonable request.
